# Is it worth allowing the presence of morning stiffness in the definition of inactive disease in juvenile idiopathic arthritis?

**DOI:** 10.1186/1546-0096-12-S1-P174

**Published:** 2014-09-17

**Authors:** Maddalena Allegra, Maria Chiara Gallo, Sara Verazza, Serena Calandra, Sara Dalprà, Federica Mongelli, Alberto Martini, Angelo Ravelli, Alessandro Consolaro

**Affiliations:** 1 Pediatria II, Istituto Giannina Gaslini, Genova, Italy; 2 Pediatrics, Università degli Studi di Genova, Italy; 3 Pediatrics, Istituto Giannina Gaslini, Genova, Italy

## Introduction

Morning stiffness is a major symptom of juvenile idiopathic arthritis (JIA) and it is usually associated with active disease. However, it is common view that children with disease quiescence may have some degrees of residual morning stiffness. The 2004 preliminary criteria for inactive disease (ID) in JIA did not include the assessment of morning stiffness, whereas the 2011 revision of the criteria has allowed the presence of morning stiffness lasting ≤ 15 minutes. However, it is still unknown whether the disease status of children with ID who have or do not have morning stiffness is comparable.

## Objectives

To compare the disease status of children with JIA who meet the 2011 revised criteria for ID and have or do not have a morning stiffness lasting ≤ 15 minutes.

## Methods

A database at the study center including 785 patients who had undergone a total of 2957 visits, which included a parent report of the presence and duration of morning stiffness, was analyzed to identify all visits in which patients met the criteria for ID. In each visit, the duration of morning stiffness was categorized as follows: ≤15 min, 15-30 min, 30-60 min, 1-2 hr, >2 hr. Clinical assessments included demographic features, and parent-reported outcomes. In case a patient met the ID criteria in more than 1 visit, only the first visit was retained.

## Results

A total of 460 visits in which the patient met the criteria for ID were evaluated. Absence of morning stiffness was reported in 390 (84.8%) visits, whereas in 70 visits (15.2%) there was morning stiffness. Among the visits with morning stiffness, in 41 (8.9%) duration was ≤15 min, and in 29 (6.3%) duration was >15 min. Table 1 shows the comparison of disease duration and parent-reported outcomes between patients with absence or presence of morning stiffness.

**Table 1:** 

	Patients meeting 2004 ID criteria			
	Patients meeting 2011 ID criteria			

	No MS N = 390	MS ≤ 15 min N = 41	MS > 15 min N = 29	p-value

Median (IQR) disease duration	3.8 (1.8; 7.3)	2.8 (1.4; 6)	5.7 (2.6; 7.9)	0.30

Functional ability (JAFS score) >0, N (%)	64 (16.4)	18 (43.9)	23 (79.3)	< 0.001

Physical health (PRQL PhS) >0, N (%)	173 (44.4)	32 (78)	28 (96.6)	< 0.001

Psychosocial health (PRQL PhS) >0, N (%)	188 (48.2)	29 (70.7)	24 (82.8)	< 0.001

VAS well-being >0, N (%)	155 (40.4)	32 (82.1)	28 (100)	< 0.001

VAS pain >0, N (%)	102 (26.8)	28 (68.3)	21 (87.5)	< 0.001

Acceptable symptom state, N (%)	366 (95.6)	33 (80.5)	16 (57.1)	< 0.001

MS: morning stiffness; IQR: interquartile range

## Conclusion

Among patients who met the 2011 criteria for ID, those with morning stiffness ≤15 min had worse parent-reported outcomes than those without morning stiffness. This finding suggests that parents may not perceive their child’s disease state as true remission when lower degrees of morning stiffness are present. Notably, a sizeable proportion (6.3%) of children meeting the 2004 ID criteria had morning stiffness lasting > 15 min. The change of the criterion “Duration of morning stiffness of ≤ 15 minutes” to “Absence of morning stiffness” in the definition for ID should be considered.

## Disclosure of interest

None declared.

